# Brain tissue stiffness is a sensitive marker for acidosis

**DOI:** 10.1016/j.jneumeth.2016.07.002

**Published:** 2016-09-15

**Authors:** Kathrin Holtzmann, Hélène O.B. Gautier, Andreas F. Christ, Jochen Guck, Ragnhildur Thóra Káradóttir, Kristian Franze

**Affiliations:** a Department of Physics, University of Cambridge, J J Thomson Avenue, Cambridge CB3 0HE, United Kingdom; b Department of Physiology, Development and Neuroscience, University of Cambridge, Downing Street, Cambridge CB2 3DY, United Kingdom; c Wellcome Trust – Medical Research Council Cambridge Stem Cell Institute, University of Cambridge, Tennis Court Road, Cambridge CB2 1QR, United Kingdom; d Biotechnology Center, Technische Universität Dresden, Tatzberg 47/49, 01307 Dresden, Germany

**Keywords:** AFM, atomic force microscopy, Acidosis, Carbon dioxide, Mechanical properties, AFM, Cerebellum, Tissue mechanics

## Abstract

**Background:**

Carbon dioxide overdose is frequently used to cull rodents for tissue harvesting. However, this treatment may lead to respiratory acidosis, which potentially could change the properties of the investigated tissue.

**New method:**

Mechanical tissue properties often change in pathological conditions and may thus offer a sensitive generic readout for changes in biological tissues with clinical relevance. In this study, we performed force-indentation measurements with an atomic force microscope on acute cerebellar slices from adult rats to test if brain tissue undergoes changes following overexposure to CO_2_ compared to other methods of euthanasia.

**Results:**

The pH significantly decreased in brain tissue of animals exposed to CO_2_. Concomitant with the drop in pH, cerebellar grey matter significantly stiffened. Tissue stiffening was reproduced by incubation of acute cerebellar slices in acidic medium.

**Comparison with existing methods:**

Tissue stiffness provides an early, generic indicator for pathophysiological changes in the CNS. Atomic force microscopy offers unprecedented high spatial resolution to detect such changes.

**Conclusions:**

Our results indicate that the stiffness particularly of grey matter strongly correlates with changes of the pH in the cerebellum. Furthermore, the method of tissue harvesting and preparation may not only change tissue stiffness but very likely also other physiologically relevant parameters, highlighting the importance of appropriate sample preparation.

## Highlights

• We introduce tissue stiffness as a sensitive indicator for pathophysiological changes to CNS tissue.
• We applied atomic force microscopy to investigate tissue stiffness.
• We found that CO_2_ overexposure-induced acidosis changes brain properties.

## 1 Introduction

Brain tissue is very sensitive to environmental changes, and particularly to a lack of oxygen. CO_2_ overdose, which is a standard method to cull rodents for tissue harvesting, leads to an impairment of the oxygen-carbon dioxide exchange, causing death by asphyxiation. It furthermore overloads the capacity of the physiological pH bicarbonate buffering system within the body. This exhaustion of the bicarbonate buffer leads to a respiratory acidosis, *i.e.*, a drop of blood pH below the physiological range of ∼7.4 ± 0.05 in mammals.

Brain tissue is protected from acidosis by the cerebrospinal fluid, which *in vivo* has a larger buffer capacity than blood (Kazemi et al., 1967). Nevertheless, while the effect of metabolic acidosis (*e.g.*, after ischemia) on brain tissue has been studied in some detail (Kraut and Madias, 2010), it is still largely unclear how the low blood pH after terminal CO_2_ overdose affects brain tissue harvested for experimental investigations. Experimental data on pure hypercapnia, obtained by ventilating animals with gas mixtures containing a high CO_2_ concentration at normoxia, have shown that brain intracellular pH may drop from a normal value of 7.04 to around 6.65 without inducing permanent damage (Siesjö et al., 1972).

Mechanical tissue properties offer a sensitive readout for chemical changes in biological tissues. Changes in tissue mechanics accompany pathological changes in different organ systems, and they can often be detected even before histological changes are visible. After the induction of liver fibrosis, for example, an increase in liver stiffness precedes the onset of classical fibrosis markers (Georges et al., 2007). Furthermore, in neurodegenerative diseases, such as Multiple Sclerosis (Streitberger et al., 2012) and Alzheimer’s disease (Murphy et al., 2011), brain tissue becomes significantly softer. In other organ systems, disease-related changes in tissue stiffness are already used in clinical diagnostics. Mechanical changes in epithelial tissues, for example, are exploited to diagnose breast cancer (Itoh et al., 2006), and an increased arterial stiffness indicates a larger risk for cardiovascular diseases (Mitchell et al., 2010).

**Fig. 1:**
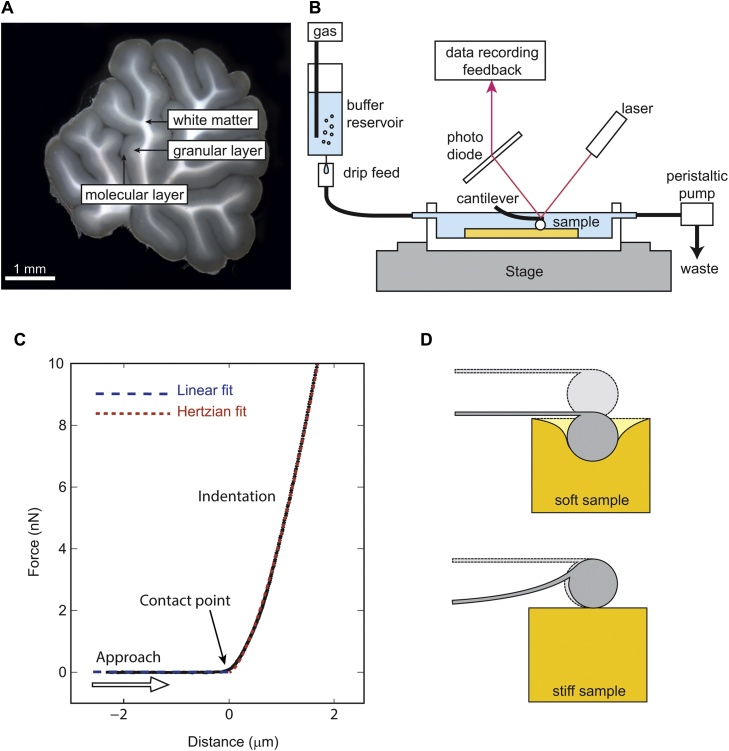
Experimental setup. (A) Bright field image of a brain slice. (B) Schematic of the AFM setup. (C) Force-distance curve of the cantilever approach (black dots). While the cantilever approaches the sample (empty arrow), deflection is zero until it gets in contact with the sample surface. The contact point (filled arrow) is determined by the intersection between a linear fit of the baseline (blue dashed line) and the Hertzian fit (red dashed line). Any subsequent downwards movement of the cantilever increases the indentation of the sample and the cantilever deflection. The latter is proportional to the applied force. (D) Schematics of indentations of very soft (sample stiffness is significantly smaller than cantilever stiffness) and stiff materials (For interpretation of the references to color in this figure legend, the reader is referred to the web version of this article.).

Thus, if respiratory acidosis leads to changes in brain tissue architecture and/or function, it is likely that these changes are accompanied by alterations in its mechanical properties. Atomic force microscopy (AFM) is well suited to detect such changes, and its high spatial resolution allows investigating specific regions in the tissue, such as white and grey matter, and even different layers within the grey matter (Fig. 1a).

During an experiment, a small leaf spring – the cantilever – is moved towards the sample until it exerts a set force. This results in the deflection of the cantilever and the indentation of the sample. Cantilever deflection is detected by a laser beam that is reflected off the cantilever’s surface onto a photodiode (Fig. 1b). After calibration, this signal is converted into a force. The AFM then records a force-distance curve, plotting the force the cantilever exerts on the sample versus its position relative to the surface (Fig. 1). The apparent elastic modulus of the sample, which is a measure of its elastic stiffness, can be calculated from force-distance curves using the Hertz model. Combining AFM with a motorized microscope stage allows tissue elasticity scans of large areas, and recording forces in the piconewton to nanonewton range with micrometer resolution.

To establish whether respiratory acidosis induced by CO_2_ overdose leads to changes in brain tissue, which might impact consecutive measurements of experimental parameters, we induced pH changes in the tissue in different ways and correlated these changes with changes in the tissue’s mechanical properties.

## 2 Materials and methods

### 2.1 Slice preparation

All procedures were performed according to the UK Animals (Scientific Procedures) Act of 1986. Animals were culled by decapitation, overdose with carbon dioxide or overdose with anesthetic (Pentobarbitone Sodium 20%w/v, Pentoject, LD50 in rats: 118 mg/kg). A power analysis based on previous measurements (Christ et al., 2010) suggested the minimum number of animals required per group to be 3.

For AFM measurements, the brain was removed and kept in ice-cold slicing solution containing 120 mM NaCl, 26 mM NaHCO_3_, 1 mM NaH_2_PO_4_, 2.5 mM KCl, 2 mM MgCl_2_, 2 mM CaCl_2_, 10 mM glucose and 1 mM kynurenic acid (a broad spectrum inhibitor of glutamate receptors used to reduce excitotoxicity), which was oxygenated with 5% CO_2_ and 95% O_2_, at pH 7,4. Immediately after brain removal, 300 μm slices were cut with a vibratome (VT1200S, Leica, Milton Keynes, UK). Slices were then transferred to a HEPES Ringer solution at pH 7.4, containing 144 mM NaCl, 2.5 mM KCl, 2.5 mM CaCl_2_, 1 mM NaH_2_PO_4_, 10 mM HEPES and 10 mM glucose. The buffer was pH adjusted and oxygenated with 100% O_2_. Slices were incubated in the HEPES Ringer solution at room temperature for an hour to allow the tissue to equilibrate.

A subset of slices was incubated in oxygenated slicing medium containing amiloride (100 mM) for 10 min and then transferred to a phosphate buffer at pH 6 containing 144 mM NaCl, 2.5 mM KCl, 2.5 mM CaCl_2_, 8.8 mM NaH_2_PO_4_, 1.2 mM Na_2_HPO_4_, 10 mM glucose and optionally 100 mM amiloride. The phosphate buffer was oxygenated with 100% O_2_. Slices were attached to plastic culture dishes (TRP, Helene labs) with BD Cell-Tak Cell and Tissue Adhesive (BD Biosciences, Franklin Lakes, USA) and held down with a harp slice grid. For each condition, we used acute slices from at least three animals.

### 2.2 pH measurements

Immediately after death, skulls were opened and a 3 mm diameter pH probe (InLab®Micro, Mettler Toledo AG, Switzerland) inserted into the cortex. The pH meter readout was recorded every 5 s for the duration of 5 min. The pH probe was calibrated immediately before each measurement and calibration was confirmed after the measurement to exclude drift.

### 2.3 Atomic force microscopy

37.5 μm polystyrene beads (microParticles GmbH, Berlin, Germany) were glued to tipless cantilevers (*k* = 0.03–0.05 N/m, Arrow-TL1, NanoWorld, Neuchatel, Switzerland) using UV curable superglue (Loctite, USA). The spring constant *k* of the cantilevers was measured before attaching the bead using the thermal noise method implemented in the AFM software (JPK Instruments AG, Berlin, Germany).

Force-distance curves were recorded with a Nanowizard III atomic force microscope (JPK Instruments AG, Berlin, Germany) under flux of oxygenated buffer at room temperature similar to electrophysiological set-ups for patch clamp experiments. Slices were indented at 0.3 Hz with a maximum force of 20 pN. The resulting force-distance curves were analyzed using the Hertz model, where

F=43E(1−v2)Rδ32

Here, *F* is the applied force, *E* is the Young’s modulus, ν is the Poisson’s ratio, *R* the radius of the probe, and δ the indentation of the sample. Approach curves were analyzed for an indentation of 3 μm as described previously (Christ et al., 2010). The reduced apparent elastic modulus *K* = *E*/(1* − ν*^2^) provides a measure of elastic stiffness: the larger *K* the stiffer a tissue (Fig. 1c). Values shown correspond to the averages of median values of tissues from individual animals ± standard deviation. By rastering over acute cerebellar tissue slices kept in oxygenated medium, hundreds of force measurements were obtained on each sample.

The averages of the median values of each animal were calculated and significance tested using One Way or Two Way ANOVA tests, followed by a post-hoc Tukey test (OriginPro 8.5, OriginLab Corporation, Northhampton, USA).

## 3 Results

**Fig. 2:**
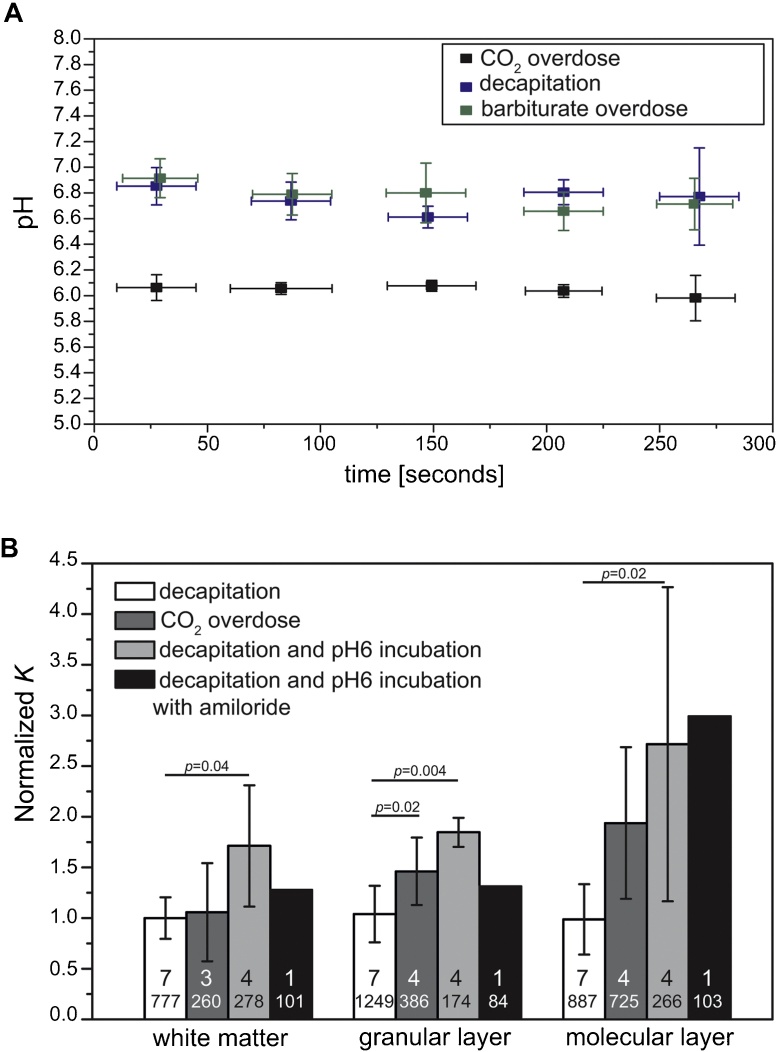
Respiratory acidosis leads to a stiffening of grey matter. (A) pH in the brain of animals culled by CO_2_ overdose, decapitation and barbiturate overdose for five minutes *post mortem*. CO_2_ overdose leads to acidosis in the brain. (B) Normalized apparent elastic modulus of different cerebellar regions after decapitation with and without subsequent incubation at pH 6, amiloride treatment as well as after CO_2_ euthanasia. Numbers of animals (top) and numbers of measurements (bottom) are shown on the bars, significance levels are indicated above the bars (one-way ANOVA followed by Tukey post-hoc test). Error bars show the standard deviation and indicate inter-animal variability.

Approved methods of euthanasia of laboratory rodents include CO_2_ overexposure, decapitation, and anesthetic overdose. Since overexposure of CO_2_ – but not the other methods – leads to a respiratory acidosis and hence increases blood acidity, we first tested if the pH of brain tissue also changes accordingly. Indeed, overdose of carbon dioxide led to a significantly decreased pH in the tissue if compared to decapitation, which is a very fast method which interferes minimally with tissue physiology, and overdose of anesthetics, which has a similar time course as CO_2_ overexposure (pH = 6.0 ± 0.02 after CO_2_ overexposure *vs.* 6.8 ± 0.04 in both decapitated and overdosed conditions over five minutes recordings; *p* < 10^−5^, Two-Way-ANOVA) (Fig. 2A). The pH trace from animals culled by an overdose of anesthetic overlapped with that of decapitated animals (*p* = 0.92), indicating that the decrease in tissue pH after CO_2_ overdose was caused by respiratory acidosis rather than by the time course of the treatment.

We then used atomic force microscopy to assess the mechanical properties of brain tissue obtained by different methods of culling. Decapitation yielded tissue with an apparent elastic modulus *K* of around 220 Pa. In animals culled by CO_2_ overdose, however, grey matter in the granular layer significantly stiffened (329 ± 75 Pa, *p* < 0.05) (*cf.* Fig. 1A). White matter, on the other hand, was not significantly affected (239 ± 109 Pa, *p* = 0.98) (Fig. 2B).

To test if the observed change in pH was sufficient to alter mechanical tissue properties, we incubated acute cerebellar slices obtained by decapitation for one hour at pH 6, thus mimicking the acidic environment caused by CO_2_ overdose (Fig. 2A). Similarly as in CO_2_-overdosed brains, grey matter significantly stiffened (612 ± 350 Pa and 416 ± 32 Pa for the molecular and granular layer, respectively), suggesting a strong correlation between alterations in the pH of the environment and tissue stiffening. Interestingly, incubation in acidic medium also affected the white matter, which stiffened significantly (386 ± 135 Pa, *p* < 0.05).

A sustained decrease in tissue pH leads to the activation of acid-sensitive ion channels (ASICs) (Waldmann et al., 1997). Opening of ASICs causes uncontrolled ion influx and cell death by excessive swelling (oncosis). These channels are predominantly found in the grey matter of the brain (Wemmie et al., 2003), where we found the tissue to mechanically change most. To test whether ASICs mediate the effect of the decreased pH on brain stiffness, tissue from decapitated animals was first incubated in slicing medium containing amiloride, and then transferred to an amiloride-containing buffer at pH 6. Amongst many functions, amiloride serves as a broad-spectrum ASIC blocker. After an incubation period of one hour, tissue stiffness was similar to that of tissue incubated in pH 6 medium without amiloride (Fig. 2B), indicating that ASICs might not drive acidosis-related changes in tissue mechanics.

## 4 Discussion

Atomic force microscopy, which we here combined with optical microscopy and a motorized stage, offers a straight-forward way to assess local mechanical tissue properties. These provide a sensitive readout of physiological and pathological changes of brain tissue, with the capability to distinguish between different brain regions. We have shown that CO_2_ overexposure, a method that is currently widely used to cull rodents, leads to changes in brain pH and in the mechanical properties of cerebellar grey matter.

Due to the insertion of an electrode into the brain parenchyma during the pH measurements, cell integrity in that region was likely compromised. Thus, the measured pH values likely represent a mixture of extracellular and intracellular pH values. The low intracellular pH in brain tissue of 7.04 (Siesjö et al., 1972) together with the normal drop in pH after culling may explain the pH values of around 6.8 for control tissue measured in this study. Importantly, a pH of 6.8 is above the threshold of 6.65, where no permanent damage occurs (Siesjö et al., 1972).

Data obtained in this study are comparable to previously published results of AFM measurements of CNS tissue mechanics, where both rat brain and mouse spinal cord tissue were found to be soft, predominantly elastic materials with apparent elastic moduli in the range of 200–400 Pa and 50–150 Pa, respectively (Christ et al., 2010, Elkin et al., 2010, Koser et al., 2015). We found that CO_2_ overdose leads to a stiffening particularly of grey matter. For example, the apparent elastic modulus of the molecular layer doubled compared to that of decapitated animals (Fig. 2B). While the standard deviation of the measured elastic moduli was <80 Pa in decapitated animals, in tissue from animals sacrificed by CO_2_ overexposure it went up to 170 Pa. Accordingly, a power analysis suggested that five samples are required to detect such differences (power of >0.8).

Grey matter stiffness increased both after CO_2_ overdose, which led to an acidification of brain tissue, and after incubation of tissue harvested from decapitated animals in an acidic solution, suggesting that acidification of brain tissue caused the observed mechanical changes. CNS tissue mechanics is mostly governed by the extracellular matrix (ECM) and individual cells (Koser et al., 2015). As the stiffening was much more pronounced in the grey matter, which in the cerebellum has the highest cell density in the whole CNS, it could be caused by a reaction of individual cells. *In vitro*, glial cells react to a drop in pH with swelling (Kempski et al., 1988). While AFM indentation on osmotically swollen primary rat astrocytes has not confirmed a stiffening of individual cells (Spagnoli et al., 2008), the swelling of the brain in a confined space could lead to an increase in pressure within the tissue, thus resulting in an increase in apparent elastic modulus. However, it is not clear if the confinement of cells in thick tissue slices as used in the current experiments would be large enough to lead to a swelling-based increase in tissue pressure.

Using amiloride as a first approach to block acid sensing ion channels (ASICs) did not have a measureable effect on tissue stiffness (Fig. 2B). Amiloride, however, has a wide range of functions, including blocking epithelial sodium channels, inhibiting cGMP-gated cation channels, and blocking sodium-hydrogen antiporters. A detailed investigation of the role of ASICs in tissue stiffening will require more closely targeted interventions.

In the CNS, ECM mainly consists of proteoglycans, hyaluronan, tenascins and link proteins. Proteoglycans are highly charged polyanions, which react sensitively to changes in ionic strength and pH. Although their conformational behaviour in biological systems is not fully understood, the pronounced increase in proton concentration in acidosis likely has an impact on electrostatic repulsion between negatively charged moieties and therefore proteoglycan conformation (Bathe et al., 2005), which might also lead to a change in tissue mechanics.

## 5 Conclusion

Our study highlights mechanical tissue properties, which are likely to change in conditions such as stroke, traumatic brain injuries and brain tumours, as a sensitive marker for pathophysiological changes in the CNS. Exploiting tissue mechanics, we have shown that CO_2_ overdose may cause biological changes in cerebellar tissue, suggesting that, depending on tissue type and the requirements of the experiment, other methods, including decapitation and anesthetic overdose, should be preferentially applied. Our work underlines the necessity for tightly controlling experimental parameters, particularly when measuring living tissue. Defining standards will be an important step to make data more comparable and reproducible in the future.
